# Molecular mechanism of Cuscutae semen–radix rehmanniae praeparata in relieving reproductive injury of male rats induced with tripterygium wilfordii multiglycosides: A tandem mass tag-based proteomics analysis

**DOI:** 10.3389/fphar.2023.1050907

**Published:** 2023-02-17

**Authors:** Shanshan Han, Yanlin Dai, Lihui Sun, Yaping Xing, Ying Ding, Xia Zhang, Shanshan Xu

**Affiliations:** ^1^ Pediatric Medical College, Henan University of Traditional Chinese Medicine, Zhengzhou, China; ^2^ Department of Pediatrics, The First Affiliated Hospital of Henan University of Traditional Chinese Medicine, Zhengzhou, China

**Keywords:** semen Cuscutae, radix rehmanniae praeparata, GTW, reproductive injury, proteomics

## Abstract

**Background:** We determined the effects of Cuscutae semen (*Cuscuta chinensis* Lam. or *Cuscuta australis* R. Br.)–Radix rehmanniae praeparata (*Rehjnannia glutinosa* Libosch.) on the protein levels in testicular tissues of rats gavaged with tripterygium wilfordii multiglycosides (GTW) and elucidated the molecular mechanism underlying Cuscutae semen–Radix rehmanniae praeparata for relieving GTW-induced reproductive injury.

**Methods:** A total of 21 male Sprague–Dawley rats were randomly divided into the control group, model group, and Cuscutae semen–Radix rehmanniae praeparata group based on their body weights. The control group was given 10 mLkg^−1^ of 0.9% normal saline by gavage daily. The model group (GTW group) was administered with 12 mg kg^-1^ GTW by gavage daily. Cuscutae semen–Radix rehmanniae praeparata group (the TSZSDH group) was administered with 1.56 gkg^−1^ of Cuscutae semen–Radix rehmanniae praeparata granules daily according to their model group dosing. The serum levels of luteinizing hormone, follicle-stimulating hormone, estradiol, and testosterone were measured after 12 weeks of continuous gavage, and the pathological lesion of testicular tissues was observed. Differentially expressed proteins were evaluated by quantitative proteomics and verified by western blotting (WB) and Real-Time Quantitative Polymerase Chain Reaction (RT-qPCR).

**Results:** Cuscutae semen–Radix rehmanniae praeparata can effectively relieve pathological lesions of GTW-induced testicular tissues. A total of 216 differentially expressed proteins were identified in the TSZSDH group and model group. High-throughput proteomics revealed that differentially expressed proteins are closely associated with the peroxisome proliferator-activated receptor (PPAR) signaling pathway, protein digestion and absorption, and protein glycan pathway in cancer. Cuscutae semen–Radix rehmanniae praeparata can significantly upregulate the protein expressions of Acsl1, Plin1, Dbil5, Plin4, Col12a1, Col1a1, Col5a3, Col1a2, Dcn, so as to play a protective role on testicular tissues. Acsl1, Plin1, and PPARγ on the PPAR signaling pathway were verified by WB and RT-qPCR experiments, which were found to be consistent with the results of proteomics analysis.

**Conclusion:** Cuscutae semen and Radix rehmanniae praeparata may regulate the PPAR signaling pathway mediated Acsl1, Plin1 and PPARγ to reduce the testicular tissue damage of male rats caused by GTW.

## 1 Introduction

Tripterygium wilfordii multiglycosides (GTW) is a metabolite of the botanical drug *Tripterygium wilfordii* Hook. f. (TW), a non-steroidal immunosuppressant. It is commonly used in Chinese traditional medicine and is known as the “Chinese herbal hormone.” It has anti-inflammatory, antitumor, and immunoregulatory effects. Therefore, GTW has been widely used for the treatment of immunological diseases, such as rheumatoid arthritis, systemic lupus erythematosus, and nephrotic syndrome. However, its reproductive toxicity has become an important factor that has limited its clinical application. Testicular diseases such as oligospermia, asthenospermia, and azoospermia occur in men, resulting in decreased fertility or even infertility (Yang. et al., 2019). Therefore, it is very important to find an effective drug that can antagonize the reproductive toxicity of GTW, so that the clinical application can exert the maximum effect without worrying about the damage to its reproductive system, and realize the “freedom of medication” for reducing toxicity and increasing efficacy.

Kidney-tonifying methods and kidney-tonifying drugs can antagonize GTW reproductive toxicity through different pathways (Sheng et al., 2017). Cuscutae semen is the dry and mature seed of *Cuscuta chinensis* Lam. or *Cuscuta australis* R. Br., which is the plant of the family Convolvulaceae. It was first recorded in *Shennong*’*s classic of botanical drugs*. It can nourish the liver and kidney, consolidate the essence, reduce urine, calm the fetus, brighten the eyes, and relieve diarrhea. Radix rehmanniae praeparata is prepared and processed product of *Rehjnannia glutinosa* Libosch.’s root tuber, which is the plant of the family Scrophulariaceae. It is an important medicine for nourishing the kidney and replenishing the blood. It is slightly warm in nature and sweet in flavor. It belongs to the liver and kidney channels and has the effect of nourishing the yin, blood, and bone marrow. Between them, one belongs to yin and another belongs to yang. They complement each other with hardness and softness and adjust the balance of yin and yang. They are Traditional Chinese Medicine (TCM) pairs in the classical kidney-tonifying formula preferred by the ancients. For example, the “Cuscutae semen Radix rehmanniae praeparata decoction” in *the Syndrome Differentiation record* has the effect of tonifying the kidney and strengthening the yang. It is used to treat kidney injury caused by excessive sexual intercourse, impotence, and premature ejaculation. The “Zuogui Pill” in *Jingyue Quanshu* can nourish yin and kidney, nourish the bone marrow, and treat spermatorrhea. Modern pharmacological studies have found that (Wang et al., 2021) metabolites of Cuscutae semen contain flavone, polysaccharides, alkaloids, steroids, terpenes, volatile oils, and lignans. The effective metabolites of Radix rehmanniae praeparata are mainly sugars, sitosterol, and nucleosides. They have anti-aging, anti-oxidation, and anti-apoptotic properties. Cuscutae semen can significantly improve sperm capillary penetration ability, sperm forward movement speed, and sperm activity index (Wang et al., 2021). Flavone, the metabolite of Cuscutae semen, can reduce the apoptosis of spermatogenic cells and increase the weights of the testis and epididymis (Meng et al., 2020). Moreover, the total flavone of Cuscutae semen can inhibit the apoptosis of testicular cells in rats and prevent oxidative damage in testicular cells. Therefore, it is generally a good curative effect for the treatment of male reproductive diseases caused by active oxygen free radicals (Zhen et al., 2006). Additionally, Yang et al, (2017) reported that Cuscutae semen polysaccharides can tonify the kidneys and support the yang. It can increase testosterone and estradiol levels, reduce blood urea nitrogen levels, improve immune function, and antioxidant effect. Zhang et al, (2012) reported that Radix rehmanniae praeparata can downregulate the GTW-induced reproductive-related spermatogenic cells gene, *c-jun*. The expression of Wnt4 is abnormal, which reduces GTW reproductive toxicity. During the early stage, we have confirmed that Cuscutae semen–Radix rehmanniae praeparata (the TSZSDH group) can not only interfere with GTW-induced injury by regulating the spermatogenic cells cycle, apoptosis, and related proteins but also has certain anti-inflammatory activity, which can effectively promote the anti-oxidation and delay cell senescence. Previous studies have shown that they can protect reproductive organs, improve sperm quality, promote testicular development, and inhibit cell apoptosis.

In this study, we showed that Cuscutae semen–Radix rehmanniae praeparata can reduce GTW-induced pathological lesions of the testicles. We further used high-throughput proteomics to investigate the ability of TSZSDH to improve the molecular mechanism of GTW-induced reproductive injury and analyzed differentially expressed proteins in testicular tissues of different groups. Then, the protein–protein interaction network and the genes and pathways affected by the treatment of TSZSDH were analyzed in the model group. Additionally, key pathway proteins were verified and clarified the therapeutic mechanism of TSZSDH in GTW reproductive toxicity.

## 2 Materials and methods

### 2.1 Reagents

**FIGURE 1:**
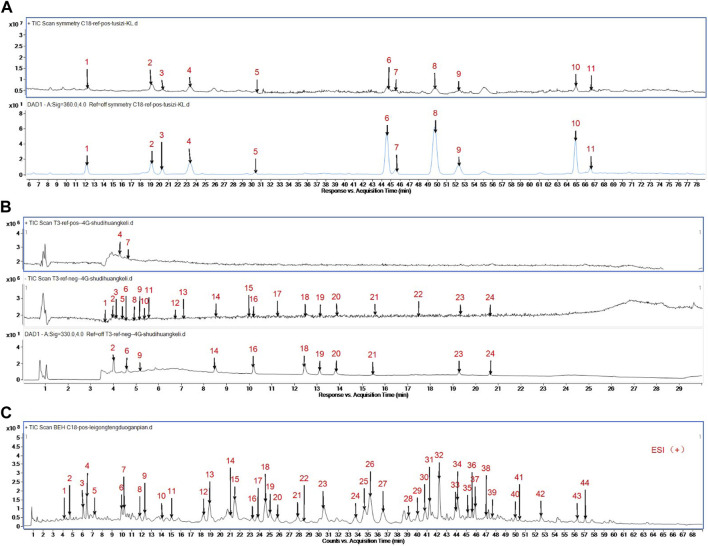
**(A)** Analysis of Prepared Cuscutae semen Dispensing Granules by Mass Spectrometry. Peak 1:Neochlorogenic acid; Peak 2:Chlorogenic Acid; Peak 3:Quercetin 3-O- β- D-galactosyl-7-O-β- D-glucoside; Peak 4:Cryptochlorogenic acid; Peak 5:Rutin; Peak 6:Quercetin-3-O-apiose - (1→2) - galactoside; Peak 7:Quercetin 3-O-β- D-galactoside (2→1)-β- D-glucoside; Peak 8:Hyperoside; Peak 9:Isoquercitrin; Peak 10:Astragalin; Peak 11:Isorhamnetin 7-O-β-D-glucopyranoside. **(B)** Analysis of Prepared Radix rehmanniae praeparata Dispensing Granules by Mass Spectrometry. Peak 1: catalpol; Peak 2: 5-hydroxymethylfurfural diglucoside; Peak 3: Rehmannin D; Peak 4: 5-hydroxymethylfurfural; Peak 5: Melittoside; Peak 6: Geniposidic acid; Peak 7: adenosine; Peak 8: Ajugol; Peak 9: 8-Epi-Loganic acid; Peak 10: Verbasoside; Peak 11: aucubin; Peak 12: rehmapicrogenin; Peak 13: Kanokoside A; Peak 14: Purpureaside C; Peak 15: Rehmaionoside B; Peak 16: Jionoside A1; Peak 17: Rehmaionoside A; Peak 18: Verbascoside; Peak 19: Jionoside B1; Peak 20: Isoacteoside; Peak 21: Cistanoside C; Peak 22: Jionoside D; Peak 23: Martynoside; Peak 24: Isomartynoside. **(C)** Analysis of Prepared GTW by Mass Spectrometry. Peak 1:Celacinnine; Peak 2:unknown; Peak 3:TripfordineA; Peak 4:unknown; Peak 5:Wilfordlongin; Peak 6:Wilforjing; Peak 7:A1atusinine; Peak 8:2-O-deacetyl-euonine; Peak 9:wilfordinine B; Peak 10:hypoglaunine E; Peak 11:1-desacetylwilfortrine; Peak 12:Tripterifordin; Peak 13:Wilfordine E; Peak 14:Wilfordinine A; Peak 15:Wilfortrine; Peak 16:Triptonoterpenol; Peak 17:wilfortrine; Peak 18:Peritassine A; Peak 19:Wilfordeonine; Peak 20:wilfordinine; Peak 21:Wilfordiuetong; Peak 22:Triptoquinone B; Peak 23:Isowilfordine; Peak 24:wilfomine A; Peak 25:9″-O-acetylwilforlrine; Peak 26:Wilforgine; Peak 27:Wilformine; Peak 28:Wilforzine; Peak 29:hypoglaunine C; Peak 30:Triptonoditerpenic acid; Peak 31:Wilfomine A; Peak 32:Wilforine; Peak 33:9″-O-acetylwilforlrine isomer; Peak 34:Triptonine B; Peak 35:6a-hydroxytriptocalline A; Peak 36:Wilforine; Peak 37:9″-O-acetylwilforlrine isomer; Peak 38:cangoronine E; Peak 39:Tripterygiumine B; Peak 40:orlhosphenic acid; Peak 41:Ebenifoline E-II; Peak 42:Celastrol; Peak 43:Wilforlide B; Peak 44:triptoquinone B.

Cuscutae semen and Radix rehmanniae praeparata were provided by Jiangyin Tianjiang Pharmaceutical Factory, China (0.5 g/bag, equivalent to 10 g of crude drugs; Batch No: 21016424, 21016134); GTW was purchased from Jiangsu Meitong Pharmaceutical (Batch No: 210101); ELISA kits for FSH, LH, E2, and T were purchased from Wuhan Elabscience Biotechnology Co., Ltd.; PPAR γ antibody and rabbit antibody were purchased from Immunoway; Plin1 antibody was purchased from Affinity Biosciences; Acsl1 antibody was purchased from Cell Signaling Technology (CST). The quality control for all the materials was validated according to the Chinese Pharmacopoeia. Our entrusted company, Jiangyin Tianjiang Pharmaceutical Co., Ltd., carried out UPLC-Q-TOF/MS analysis to characterize the Cuscutae semen, Radix rehmanniae praeparata, and GTW extract (Figures 1A–C). The chemical constituents of Cuscutae semen, Radix rehmanniae praeparata, and GTW extract were profiled using an ultra-high-performance liquid chromatography system coupled with a high-resolution mode.

### 2.2 Animals

Twenty-one 4-week-old male Sprague–Dawley rats weighing 51–75 g were purchased from Beijing Weitong Lihua Co., Ltd. [License: SCXK (Beijing) 2016–0011]. The experimental protocol was approved by the Animal Experiment Ethics Committee of the Henan University of Chinese Medicine (No: DWLL202105053). The male rats were randomly assigned to the normal saline group (the control group), the GTW group (the model group), and GTW and Cuscutae semen–Radix rehmanniae praeparata treatment group (the TSZSDH group), with seven rats in each group fed according to the 12-h light/dark cycle.

### 2.3 Medicine dose and model preparation

Rats in each group were fed the same diet and administered by gavage once every morning. The control group was fed with 10 mL kg^-1^·d^-1^ of 0.9% normal saline *via* gavage. The model group was fed with a solution of 1 mL normal saline containing 1.2 mg GTW, administered *via* gavage at the dose of 10 mL kg^-1^·d^-1^ (i.e., 12 mg kg^-1^·d^-1^). The TSZSDH group: based on the model group, this group was fed with 1 mL of normal saline containing 0.156 g of Cuscutae semen and 0.156 g of Radix rehmanniae praeparata granules, administered *via* gavage at the dose of 10 mL kg^-1^·d^-1^. The rats were fed continuously for 12 weeks and weighed weekly. After fasting for 12 h, three groups of rats were anesthetized with pentobarbital and their blood was sampled from the abdominal aorta. The serum was collected, and the level of sex hormones was detected by using ELISA kits. The left epididymis was collected to detect the density and viability of sperm. The left testicular tissues were stained with hematoxylin and eosin to observe the pathological changes. The right testicular tissues were frozen at −80°C in the refrigerator for proteomic analysis.

### 2.4 Polypeptide sample preparation and TMT labeling

The right testicular tissues of each rat were stored at −80°C and accurately weighed to 50 mg, followed by the addition of the lysate (1.5% SDS/100 mM Tris-Cl, pH 8.0). After tissue homogenization in a boiling water bath, the tissues were sonicated on an ice water bath for 10 min and then centrifuged several times. The supernatant was collected and the protein in the solution was precipitated *via* acetone precipitation. Then, the complex solution, dithiothreitol (DTT), and iodoacetamide (IAA) were added to the protein precipitate until the final concentration of 40 mM was obtained, and the alkylation reaction was triggered at room temperature in the dark. The protein concentration was determined by using the Bradford method. After protein quantitative, 50 μg of the samples were taken in EP tubes for SDS-PAGE detection, and the protein bands were observed after Coomassie brilliant blue staining. To this, 100 mM of Tris-HCl solution (pH 8.0) was added to the reduced and alkylated samples, the urea concentration was diluted to <2 mM, and trypsase was added for digestion according to the mass ratio of enzyme to the protein of 1:50. An equal amount of samples was taken for TMT labeling. The marking operation was conducted in accordance with the instructions of the TMT manufacturer. After the labeled samples were mixed in an equal amount, they were desalted using the Sep-Pak C18. After vacuum drying, the mixed samples were separated by high pH reverse chromatography and finally combined into 15 components. The labeled samples were analyzed by high-resolution Orbitrap LC-MS/MS before data analyses to determine the relative abundance of the peptides. Then, 100 μg of the peptides were taken for each sample.

### 2.5 Western blotting analysis

The testicular tissues (80 mg) were extracted and crushed, followed by rinsing twice with PBS. The tissues were then homogenized and lysed with RIPA buffer containing a mixture of protein enzyme inhibitors. The protein was centrifuged at 12000 rpm for 10 min, and the protein concentration was determined by using BCA kits. Then, about 60 μg of the protein sample was extracted and separated by SDS-PAGE gel and transferred onto the PVDF membrane. Then, 5% skim milk powder was added for blocking for 1 h and then incubated with the primary antibody at 4°C overnight. After rinsing with TBST thrice (5 min each time), the membrane was incubated with secondary antibodies (anti-rabbit IgG 1:10000) for 30 min. Finally, the ECL solution was added for development.

### 2.6 Real-time quantitative polymerase chain reaction

Total RNA was obtained using TRIzol reagent (Invitrogen, U.S.A.). cDNA was obtained by reverse transcription using a Reverse Transcription Kit (TOYOBO, China) and then was amplified by RT-qPCR using 2X universal SYBR Green Fast qPCR Mix (ABclonal, China). In this study, β-actin was used as the housekeeping gene and 2^−ΔΔCT^ was used to calculate the relative change in gene expression. The primer sequences used to amplify PPARγ, Acsl1, Plin1 and β-actin are listed in Supplementary Table S1.

### 2.7 Statistical analysis

SPSS 26.0 software was used to statistically process the data, and the results were expressed as $\bar{x}\pm S$ . Based on whether the data conformed to the normality test, a non-parametric test was adopted. The research data involved the mean comparison among multiple groups, and one-way ANOVA was applied for further pairwise comparison. *p* < 0.05 was considered to indicate a statistically significant difference. The differentially expressed proteins between the model and control groups were called DEPs1 and those between the TSZSDH and model groups were called DEPs2. The screening conditions for the differentially expressed proteins were as follows: the difference fold change >1.2 times (upregulation >1.2 times or downregulation <0.83) and *p* < 0.05.

Metascape (https://Metascape. Org/gp/index. Ht mL) and eggnog were applied for Gene Ontology (GO) enrichment analysis and gene: Kyoto Encyclopedia of Genes and Genomes (KEGG) pathway enrichment analysis. Bioinformatics online website, Cytoscape3.8.1, Origin 2021, and GraphPad Prism nine software were used for graphic display. *p*-value score and gene ratio were adopted to select the important GO enrichment analysis, KEGG pathway, and COG annotation.

## 3 Results

### 3.1 Effects of Cuscutae semen–radix rehmanniae praeparata on the testis and epididymis indexes of rats in the GTW group

The rats in the control group, model group, and TSZSDH group had the same body weight before administration. Compared with the control group, the weight of the model group rat did not change significantly after 12 weeks; compared with the model group, rats in the TSZSDH group exhibited increased body weights, while the differences were no statistical significance (Supplementary Table S2). Compared with the control group, rats in the model group showed decreased testis and epididymis indexes, but the differences were not statistical significance compared with the model group. Rats in the TSZSDH group showed increased testis and epididymis indexes, but the differences were not statistical significance (Supplementary Table S2).

### 3.2 Effects of Cuscutae semen–radix rehmanniae praeparata on serum level of sex hormone of rats in the GTW group

After 12 weeks of treatment, only T changed significantly in the three groups, whereas LH, FSH, and E2 had no statistical difference. Compared with those in the control group, rats in the model group showed decreased LH, FSH, E2, and T, but the differences were not statistical significance compared with the model group. Rats in the TSZSDH group rat showed decreased LH and increased FSH, E 2, and T. The differences in T were statistically significant and the differences in FSH and E2 were not statistical significance (Supplementary Table S3).

### 3.3 Effects of Cuscutae semen–radix rehmanniae praeparata on testis and epididymis histopathology of the GTW group

The seminiferous tubule is the main component of the testis and the place organ where sperm is generated. Therefore, the diameter of the seminiferous tubule of the three groups of rats was measured and the number of seminiferous tubules was counted in their area. Compared with the control group, the area of the seminiferous tubule of the model group was shorter, but the differences were not statistical significance. Compared with the model group, the area of the seminiferous tubule of the TSZSDH group was shorter, but the differences were not statistical significance.

**FIGURE 2:**
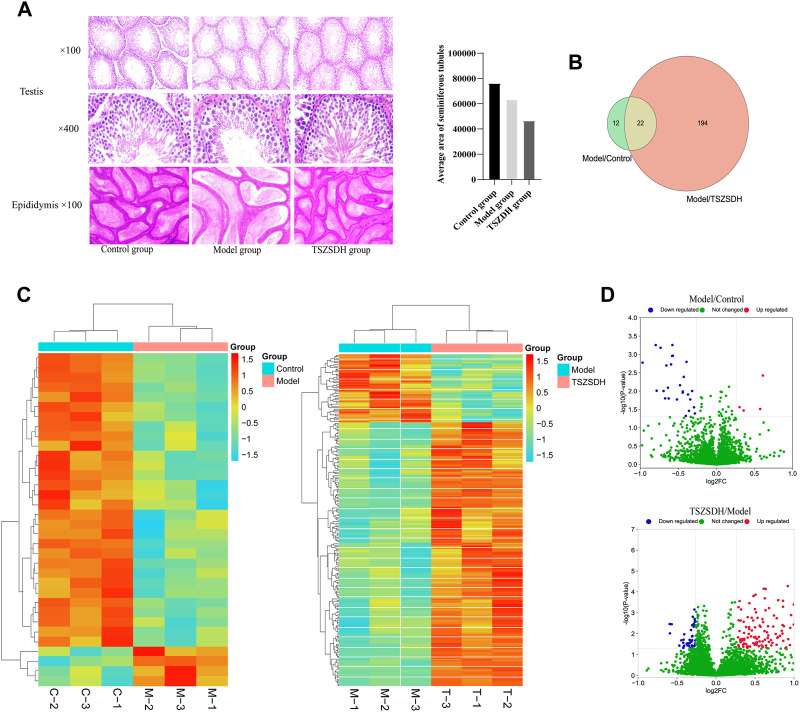
**(A)** Testicular and epididymal tissues were stained with hematoxylin and eosin (HE) and observed under a light microscope at ×100 and ×400 magnifications. Control group (Control); *Tripterygium wilfordii* multiglycosides-induced reproductive injury model group (Model); Cuscutae semen–Rehmannia Glutinosa treatment group (TSZSDH). **(B)** Results of Tandem Mass Tag (TMT) protein labeling experiments. The green part represents the results of differential protein labeling between the model and control groups; the red part represents the result of the differential protein labeling between the TSZSDH and model groups, and the crossed part in yellow represents the overlapping portion. **(C)** Clustering heat map comparing the model group with the control group, and the TSZSDH group with the model group. Upregulated protein expression is indicated in red, while downregulated protein expression is indicated in blue. **(D)** Volcano plot comparing the model and control groups, and the TSZSDH and model groups. The log2fold-change (FC) value is the abscissa and the -Log10(*p*-value) is the ordinate. UP Red indicates that differentially expressed proteins are upregulated, while blue indicates downregulated proteins, and green indicates proteins whose expression was not significantly changed.

The histopathological changes of testis and epididymis of the rats in the three groups were observed by hematoxylin-eosin staining to determine the protective effect of Cuscutae semen–Radix rehmanniae praeparata on the damage of the reproductive organs in the GTW group. The diameter of the seminiferous tubule of the rats in the model group was smaller, the space between tubes was larger, the seminiferous epithelium was thinner, the number of spermatogenic cells at all levels was significantly reduced and scattered, and the spermatogenic cells appeared vacuolization, the Leydig cells were reduced, and the number of sperms in the lumen of the epididymis tube was decreased. The epithelia of the seminiferous tubule in rats in the TSZSDH group were slightly thinner, the cells were distinct, and there was no obvious change in the intercellular space. The number of spermatogenic cells in all levels of the TSZSDH group was significantly higher than that of the model group. The intercellular space was slightly larger, the change of interstitial cells was not distinct, and the sperms in the epididymis were relatively dense (Figure 2A).

### 3.4 Effects of Cuscutae semen–radix rehmanniae praeparata on the protein expressions in testicular tissues of rats in the GTW group

**FIGURE 3:**
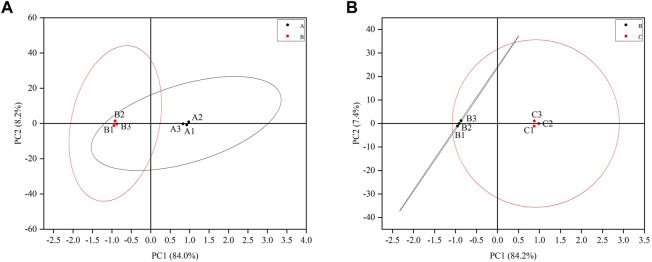
**(A)** Testicular PCA scores of male rats between the control and model groups. **(B)** Testicular PCA scores of male rats in the treatment and model groups. The letter A represents the control group, the letter B represents the model group, and the letter C represents the TSZSDH group.

A total of three testicular samples from each group were used for proteomics analysis. Through PCA analysis, indicating that the samples in each group had good repeatability, and the samples between groups had significant differences (Figure 3). Among them, the control group is mainly located in the second and fourth quadrants, the GTW group is mainly located in the first and third quadrants, and the TSZSDH group is similar to the control group, mainly located in the second and fourth quadrants. PCA results showed that the samples of rats in the control group and the GTW group were obviously separated, indicating that the samples of rats in the GTW group were significantly changed, and the samples of rats in the TSZSDH group and the GTW group were obviously separated, similar to the control group, indicating that the rats in the TSZSDH group were significantly improved after the intervention of TSZSDH. A total of 7381 proteins were identified in three groups. The difference fold change >1.2 times (upregulation >1.2 times or downregulation <0.83) and *p* < 0.05 were used as the screening criteria for differentially expressed proteins. The number of differentially expressed proteins between groups was compared. Compared with the control group, the model group had 34 statistically significant differentially expressed proteins, which included four upregulated proteins and 30 downregulated proteins. Compared with the model group, the TSZSDH group was treated with Cuscutae semen–Radix rehmanniae praeparata. A total of 216 differentially expressed proteins with statistical significance were present, which included 172 upregulated proteins and 44 downregulated proteins. The statistics of protein quantitative results are shown in the form of a volcano map and cluster heat map as shown in figures (Figures 2C,D).

### 3.5 GO term enrichment, KEGG pathway enrichment analysis, and orthologous clustering (COG) annotation analysis

**FIGURE 4:**
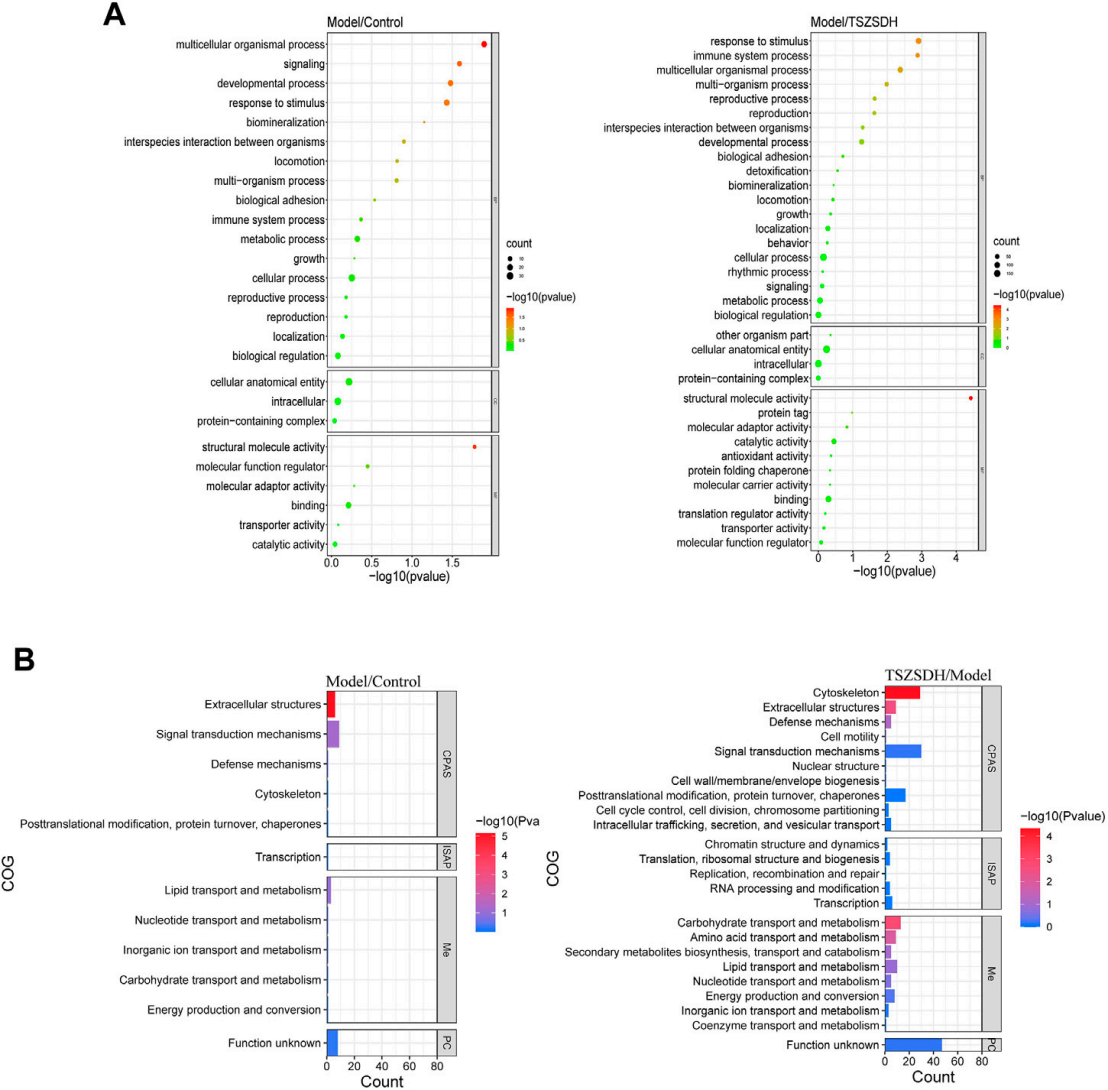
**(A)** GO terms comparison between the model and control groups, and between the TSZSDH and model groups. The size of the dots represents the number of gene proportions, and the color represents the *p*-value. The greener the color, the smaller the -log10(*p*-value), and the redder the color, the greater the -log10(*p*-value). **(B)** COG annotations comparison between the model and control groups and the TSZSDH and model groups. The length of the bar graph represents the number of gene proportions, and the color represents the value. The bluer the color, the smaller the -log10(*p*-value), and the redder the color, the larger the -log10(*p*-value).

The DEPs one of the model group and the control group and DEPs two of the TSZSDH group and the model group were subjected to GO term enrichment analysis, which included biological processes, cell components, and molecular functions. A total of 36 GO enrichment functions (bubble chart) were present between DEPs one and DEPs 2, among which 9 were pathways with *p* < 0.05. Compared with the control group, the differentially expressed proteins of the model group were significantly regulated (*p* < 0.05) in response to stimulus (11%), multicellular organismal process (11%), developmental process (9%), signaling (7%), and structural molecule activity (12%), in molecular functions while cellular anatomical entity (46%) and intracellular (41%) were regulated in cellular components and protein-containing complex (13%), but the differences were no statistical significance. Compared with the model group, the differentially expressed proteins of the TSZSDH group were significantly regulated in response to stimulus (11%), which belongs to biological processes, multicellular organismal process (9%), immune system process (4%), multi organism process (4%), reproductive process (3%), reproduction (3%), and structural molecule activity (9%) in molecular functions, whereas cellular anatomical entity (46%) and intracellular (41%) were regulated in cellular components, protein-containing complex (12%), other organic parts (0.3%), but the differences were no statistical significance. The results showed that TCM can be associated with biological functions such as response to stimulation, cell organism process, immune system process, and multi-tissue process to increase the efficacy of the treatment and play a therapeutic role (Figure 4A).

**FIGURE 5:**
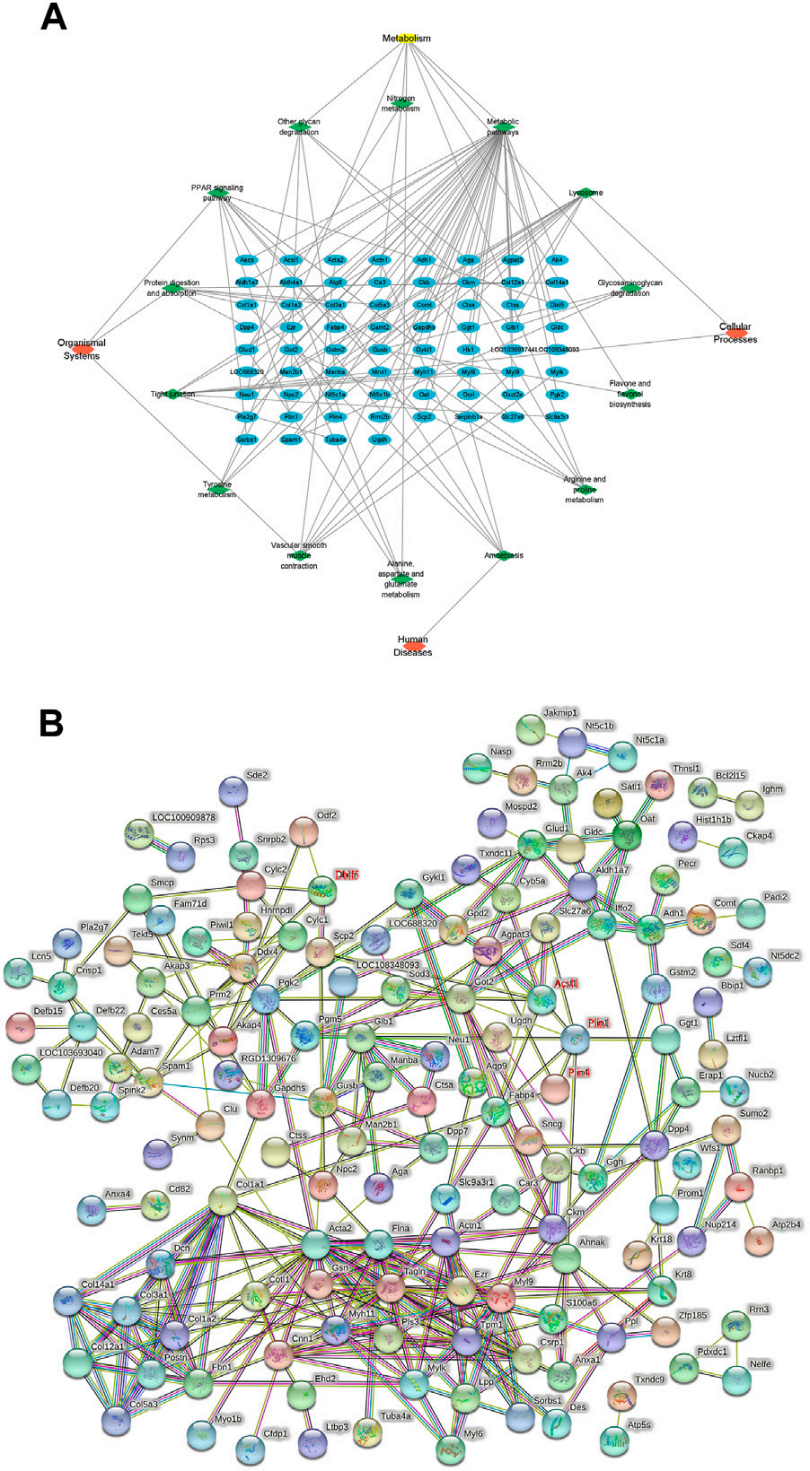
**(A)** KEGG pathway network diagram of the treatment and model groups. The blue graphs represent DEPs, the green graphs represent pathways, and the red graphs represent the types of pathways. **(B)** PPI analysis between differentially expressed proteins.

KEGG pathway analysis was performed on all DEPs, and 23 pathways with *p* < 0.05 were screened out. The DEPs in the model and control groups were enriched in the following 11 pathways: focal adhesion (50%), quorum sensing (17%) related to cellular processes, the transforming growth factor-beta signaling pathway (23%), extracellular matrix (ECM)–receptor interaction (23%) in environmental information processing, proteoglycans in cancer (25%), the advanced glycation end products (AGE)-receptors for AGE signaling pathway in diabetic complications (17%), fatty acid degradation (9%), fatty acid metabolism (9%), protein digestion and absorption (23%), the PPAR signaling pathway (19%), and vitamin digestion and absorption (4%). The DEPs in the model and TSZSDH group were enriched in the following 14 pathways: lysosome (20%) and tight junction (14%) in cellular processes, amoebiasis (6%) in human diseases, metabolic pathways (21%), other glycan degradation (3%), arginine and proline metabolism (3%), glycosaminoglycan degradation (2%), tyrosine metabolism (2%), alanine, aspartate, and glutamate metabolism (2%), nitrogen metabolism (1%), flavone and flavanol biosynthesis (0.6%) in metabolism, the PPAR signaling pathway (11%), protein digestion and absorption (9%), and vascular smooth muscle contraction (8%) in organismal systems. A network of KEGG pathways and genes was constructed using Cytoscape (Figure 5A).

COG annotation analysis was performed on all DEPs. These DEPs were functionally classified into four types (see the bubble chart): cell processes and signaling, information storage and processing, metabolism, and poorly characterized. Four pathways were statistically significant (*p* < 0.05). The DEPs in the model and control groups were enriched in one pathway related to extracellular structures (18%). The pathways enriched by the DEPs in the model and TSZSDH groups were cytoskeleton (29%), carbohydrate transport and metabolism (13%), extracellular structures (9%), and amino acid transport and metabolism (9%) according to the number of relevant genes (Figure 4B).

### 3.6 Protein–protein interaction (PPI) analysis

The relationship between the model and TSZSDH groups was determined using the STRING database to further clarify the crosstalk between molecular mechanisms and DEPs. A total of 207 nodes (4 targets without corresponding genes and five fewer in the graph shown by STRING) and 333 edges were connected. Acsl1, Plin1, Plin4, and Dbil5 occupied the central position of the PPI network, acting as a hub to interact with other DEPs (Figure 5B).

Under the screening criteria of *p* < 0.05 and differential multiple (upregulated >1.2 and downregulated <0.83), the expression of DEPs in the model group/the control group in the TSZSDH group was analyzed. DEPs in the model group were upregulated, whereas one protein was downregulated in the TSZSDH group. DEPs in the model group were downregulated, whereas 20 proteins were upregulated in the TSZSDH group. One protein was upregulated in both groups. Thus, 21 abnormally expressed proteins in the model group were corrected in the TSZSDH group (see Supplementary Table S4; Figure 2B). A total of 22 proteins were involved in the model group/the control group and the TSZSDH group/the model group (One of the upregulated proteins failed to find the gene in UniProt). The KEGG pathway enrichment analysis of these DEPs revealed that they were enriched in the PPAR signaling pathway, protein digestion and absorption, and proteoglycans in cancer (see Supplementary Table S5).

### 3.7 WB and RT-qPCR analysis

**FIGURE 6:**
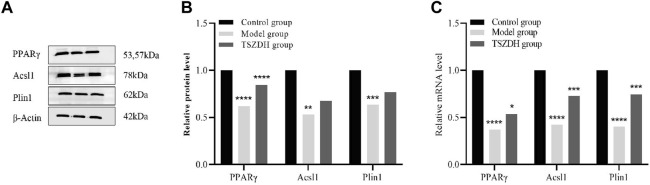
**(A)** The protein expression level of PPARγ, Acsl1, and Plin1in the testicular cells. **(B)** The relative level of the proteins in the testicular cells. **(C)** The mRNA level of PPARγ, Acsl1, and Plin1 in the testicular cells. ^*^ *p* < 0.05, ^**^ *p* < 0.01, ^***^ *p* < 0.001, ^****^ *p* < 0.0001.

The related pathways mediated by the PPAR signaling pathway were studied by the proteomics approach. Three DEPs, namely, Acsl1, Plin1, and PPARγ involved in the PPAR signaling pathway were validated by the WB and RT-qPCR method. Both experimental results are consistent. These DEPs were significantly downregulated in the model group and upregulated in the TSZSDH group (Figure 6), which was closer to the control group.

## 4 Discussion

GTW is a lipid-soluble mixture mainly extracted from TW and has biological activities such as anti-infection and immunity enhancement (Wang et al., 2022). However, gonadal damage is an obstacle to using this drug. In 1981, researchers reported that GTW could be widely used to treat kidney diseases in adults and children. Although its clinical effect is ideal, many researchers have reported its reproductive injury. Li et al. (2020) found that GTW can reduce sperm number, increase sperm-deformity rate, and cause sperm kinetic parameter abnormality. Fan et al. (2020) analyzed and summarized research on the reproductive toxicity of TW over the years. The total incidence of reproductive toxicity in patients taking TW was about 18%. The occurrence of toxicity was related to the dose, time, and combination of drugs, age, and gender of patients. Specifically, the incidence of reproductive toxicity in males was higher than that in females. The drug dose required by male drug users for a reproductive toxicity reaction was smaller than that required by females, and the time of toxic symptom appearance was also faster than that in females. Damage to the male reproductive system mainly occurs in patients with rheumatoid arthritis and skin diseases treated with TW. Its clinical manifestations mainly include reduced sperm activity, oligospermia or azoospermia, and reduced fertility or infertility. Long-term medication may also cause testicular atrophy and decreased sexual desire (Yu, 1983; Xu et al., 2019). The study results have shown that GTW can cause the atrophy of the seminiferous tubule of testicular tissues of rats, thinning of the tube wall, reduction of spermatogenic cells at all levels, and reduction of sperm in the epididymal tube lumen. However, after the intervention of Cuscutae semen–Radix rehmanniae praeparata, pathological changes in testicular tissues can be significantly improved, which is consistent with the results of proteomics and WB.

Many scholars have thoroughly studied TCM because of its advantages such as multi-component, multi-target, and multi-pathway medication. Cuscutae semen and Radix rehmanniae praeparata are TCMs for tonifying the kidney. They can act on the hypothalamus-pituitary gonad axis to exert their effects. Among them are Cuscutae semen supplements yang and Radix rehmanniae praeparata supplements yin. Combining the two can strengthen and protect the “foundation of the whole body,” “nourish the source, and worship the vitality,” and nourish both yin and yang. It coincides with the theory of TCM that “those who are good at tonifying yang must seek yang in yin” and “those who are good at tonifying yin must seek yin in yang,” which embodies the idea that yin and yang are mutually correlated. The animal experiments in this study showed that Cuscutae semen–Radix rehmanniae praeparata could significantly improve GTW-induced testicular tissues damage, repair the structure of the seminiferous tubule, increase the number of spermatogenic cells at all levels, and increase the number of sperms significantly. The high-throughput proteomics analysis revealed differential expressions of Acsl1, Plin1, Dbil5, Plin4, Col12a1, Col1a1, Col5a3, Col1a2, and Dcn, which are associated with the PPAR signaling pathway, protein digestion and absorption, and the protein glycan pathway in cancer. Herein, the amino acid sequence of a protein in the PPAR signaling pathway is highly conserved among different species, suggesting that they have more than 80% amino acid in rats, mice, and humans. Therefore, PPAR signaling pathways have attracted great attention (Bensinger and Tontonoz, 2008).

The PPAR signaling pathway protein is a member of the transcription factor superfamily of intranuclear receptors playing a key role in lipid and energy metabolisms. It controls the expression of many genes related to fatty acid input and oxidation and is closely related to glycolipid energy metabolism, oxidative stress, inflammation, cancer, and autophagy (Wang, 2010; Oyefiade et al., 2019).

PPAR comprises PPARα, PPARβ/δ, and PPARγ, which are homologous (Dubois et al., 2017). Among them, PPARα is the most widely distributed and mainly distributed in tissues rich in mitochondria, such as liver, skeletal muscle, and kidney tissues. It regulates the transport of fatty acids into mitochondria by regulating the expression of Acsl and CPT-1 to promote the β-oxidation of fatty acids in mitochondria, reducing the intracellular fatty acid levels (Singh et al., 2016). PPARβ is also widely present in various tissues; however, its physiological effect is unknown (Lefebvre et al., 2006; Wagner and Wagner, 2010). PPARγ is highly present in adipose tissues and mainly participates in fat differentiation. It can also increase the levels of the fatty acid transport protein, fatty acid transferase FAT/CD36, adipocyte-type fatty acid binding protein, phosphoenolpyruvate carboxykinase, and ACSL1, as well as enhance the expression of genes that promote fatty acid storage (Reddy RC., 2008). Simultaneously, it can reduce cholesterol and blood lipids (Reddy, 2008). Regueira et al. (2014) reported that PPARα and PPARβ/δ participate in fatty acid catabolism and glucose metabolism of Sertoli cells by promoting the β-oxidation of fatty acids to provide sufficient energy to Sertoli cells. Minutoli et al. (2009) reported that a PPARβ/δ agonist promotes the expression of the PPARβ/δ gene, which subsequently inhibits the damage of testicular tissues caused by ischemia-reperfusion injury. A PPAR agonist is widely used to treat several diseases including metabolic, chronic inflammatory, immunological, neurological, and psychiatric diseases, infections, and malignant tumors.

Currently, PPARγ is most studied by scholars. PPARγ is a ligand-dependent transcription factor in the nuclear receptor superfamily and a major regulator of the growth and development of adipose tissues (Martin and Parton, 2006; Puri et al., 2007). PPARγ has multiple biological functions and is important for regulating metabolism, controlling inflammation, improving atherosclerosis, inhibiting tumors, and regulating immune processes (Yang et al., 2022). PPARγ activation can also inhibit matrix production and reduce oxidative stress response, thus protecting tissue repair (Marder et al., 2013).

ACSL1 is present in the liver and adipocytes, and its gene is abundantly expressed in lipid droplets (LDs), microsomes, and mitochondria. As a common target gene of PPARα and PPARγ, it plays an important role in lipid and fatty acid metabolism (Fortis-Barrera et al., 2020; Grevengoed et al., 2015). ACSL1 was located in the endoplasmic reticulum, mitochondria-related membrane, and cytosol using isotype-specific antibodies but not in other components of mitochondria (Yin et al., 2022). ACSL1 is thought to play an important role in activating fatty acids to synthesize triacylglycerols (TGs). The high expression of the ACSL1 gene reduces the β-oxidation of fatty acids *via* the PPARγ pathway, thereby increasing triglyceride levels (Liu et al., 2020). Some studies have confirmed that ACSL1 is the target gene of PPARα and is regulated by PPARα. The expression of the ACSL1 gene was significantly upregulated in the mouse liver and kidney after administering a PPARα agonist (Singh et al., 2016), suggesting that Cuscutae semen–Radix rehmanniae praeparata has a similar effect on the PPARα agonist, which can improve the energy metabolism and oxidative stress of cells and inhibit the inflammatory reaction.

Plin1 is the most important member of the LD surface protein family, which is located in various tissues including liver and muscle tissues. It is also a phosphorylated protein present in rat epididymal adipocytes. It is specifically present on the surface of neutral LDs in adipocytes. It has a bidirectional regulatory role in regulating the accumulation and hydrolysis of TG and has biological functions such as regulating autophagy and inflammatory responses (Itabe et al., 2017; Zhang et al., 2018; Sun et al., 2022). Studies on mammals revealed that the transcription of the Plin1 gene is mainly regulated by PPARγ, the main regulator of adipogenesis. In mammals, PPARγ can regulate the transcription of the Plin1 gene by binding to the functional response element of PPARγ located in the 5′“flanking” region of the Plin1 gene (Arimura et al., 2004). In the basal or resting state, Plin1 protects lipids in LD from interacting with adipose triglyceride lipase (ATGL), hormone-sensitive lipase (HSL), and binds to comparative gene identification-58, which is an activator of ATGL, to reduce ineffective lipolysis, thus maintaining intracellular TG levels. In contrast, catecholamines phosphorylate Plin1, HSL, and CGI58 by activating the cyclic AMP/protein kinase A signaling pathway during eating or exercise. After phosphorylation, Plin1 dissociates from CGI58, enhances ATGL activity, and recruits phosphorylated HSL on the surface of LD, promoting the cascade decomposition of TG (Feng et al., 2021). In summary, Plin1 controls lipid turnover *in vivo* by regulating lipolysis and lipid synthesis and maintains the homeostasis of lipid metabolism.

The main active component of GTW is triptolide. Previous studies found that the testicular oxidation and inflammatory reaction of GTW-induced reproductive injury in male rats are related to the severity of the disease, inhibiting oxidation can reduce the degree of testicular inflammation injury in model animals (Singla and Challana, 2014; Sheng et al., 2017; Tan et al., 2018). PPAR levels were significantly decreased in the testicular tissues of mice treated with triptolide. Ma et al, (2015) reported that triptolide might cause the metabolic disorder of fatty acid metabolism by downregulating the expression of the PPAR gene, causing considerable changes in a group of endogenous metabolites in the testis and serum, leading to abnormal testicular lipid and energy metabolism and thus leading to dyszoospermia. PPAR and associated fatty acid metabolism may be potential targets for the intervention or treatment of triptolide-induced male infertility. PPAR may be one of the key regulators of these intermediate metabolites in Sertoli cells. Triptolide may cause abnormal levels of small-molecule metabolites in Sertoli cells by regulating PPAR, leading to dyszoospermia and male infertility (Cheng et al., 2018; Ma et al., 2015). Hence, as the main effective component of GTW, triptolide can reduce the levels of related proteins in the PPAR pathway, thus leading to abnormal lipid energy metabolism and abnormal metabolites *in vivo* and promoting oxidation, eventually causing dyszoospermia and even infertility in males.

The results of the present study suggest that GTW may downregulate the expression of the PPARγ, Acsl1, and Plin1 genes, causing the metabolic disorder of fatty acid, inhibiting energy metabolism in the testis, aggravating the oxidative stress of cells, and leading to the atrophy of the seminiferous tubule and the decrease in sperm number and vitality. Cuscutae semen–Radix rehmanniae praeparata can improve lipid energy metabolism, reduce oxidative stress, and increase sperm quantity and activity. The levels of Col12a1-, Col1a1-, Col1a2-, and Col5a3-related proteins in the model group were decreased. TSZSDH could significantly improve the levels of the related proteins. The testicular ECM includes the basement membrane of the seminiferous tubule and the intercellular matrix of peritubular cells, mainly including Type Ⅰ and Ⅳ collagens, laminin, arrestin, and protein polysaccharides, which play an important role in the self-renewal of spermatogonial stem cells and sperm generation (Yuan et al., 2017). In the KEGG pathway, Col12a1, Col1a1, Col5a3, and Col1a2 were enriched in the protein digestion and absorption pathway, suggesting that Cuscutae semen–Radix rehmanniae praeparata can promote the generation of testicular ECM, thereby improving GTW-induced reproductive injury in male rats.

## 5 Conclusion

Cuscutae semen–Radix rehmanniae praeparata can effectively relieve GTW-induced reproductive injury. The pathway enrichment analysis of DEPs indicated that the PPAR signaling pathway, protein digestion and absorption, and the protein glycan pathway in cancer play an important role in relieving this injury. Acsl1, Plin1, and PPARγ were verified by WB and RT-q-PCR experiments. Cuscutae semen and Radix rehmanniae praeparata may regulate the PPAR signaling pathway mediated Acsl1, Plin1 and PPARγ to reduce the testicular tissue damage of male rats caused by GTW.

## 6 Limitations and outlook

In this study, we used quantitative proteomics as a tool to combine TCM with *in vivo* metabolites and their pathways, which are conducive to the standardization of TCM and show the mechanism of TCM. However, this study has several limitations as follows: 1) The sample size of this study was relatively insufficient, which may lead to some experimental results without obvious statistical significance. 2) The sample sizes of the included studies were small, and the number of DEPs was less than that reported in foreign studies. 3) Although WB experiments were performed to verify DEPs in this study, only three DEPs were verified because of limited funds. 4) No other research method was followed to compare the downstream metadata of the screened DEPs with the metadata of previous studies.

Owing to the high diversity of effective components of TCM, understanding their specific mechanisms requires further clarification despite identifying some specific differential proteins and pathways. Large-sample studies are required to enhance the reliability of the study conclusions and achieve more significant results. In the present study, the metabolites of DEPs were verified by immunohistochemistry and other methods, and the effects of Cuscutae semen–Radix rehmanniae praeparata on DEPs were further discussed.

## Data Availability

The datasets presented in this study can be found in online repositories. The names of the repository/repositories and accession number(s) can be found below: iProx (http://www.iprox.org/), IPX0004150001.

## Abbreviations

GTW, tripterygium wilfordii multiglycosides; Cuscutae semen–Radix rehmanniae praeparata, TSZSDH; TW, Tripterygium wilfordii Hook.f.; WB, Western blotting; RT-qPCR, Real-Time Quantitative Polymerase Chain Reaction; TCM, Traditional Chinese Medicine; DEPs, differentially expressed proteins; TMT, tandem mass tag; PPI, protein–protein interaction; KEGG, Kyoto Encyclopedia of Genes and Genomes; GO, gene ontology; COG, cluster of the orthologous group; PLIN1, Perilipin1; ACSL1, long-chain acyl-Co A synthetases; PPARγ, peroxisome proliferator-activated receptor γ.
