# Supplementary material for: Molecular mechanism of Cuscutae semen–radix rehmanniae praeparata in relieving reproductive injury of male rats induced with tripterygium wilfordii multiglycosides: A tandem mass tag-based proteomics analysis
Source: Front Pharmacol. 2023 Feb 17;14:1050907. doi: 10.3389/fphar.2023.1050907 (PMC9982038; doi:10.3389/fphar.2023.1050907)
Supplement: Supplementary file 1 [file Table1.docx]

| **Table 1** Primer sequences used for RT-qPCR. | |
| --- | --- |
| **Gene** | **Primer sequence (5' to 3')** |
| PPARγ | Forward:TCCCGTTCACAAGAGCTGAC |
|  | Reverse:ATAATAAGGCGGGGACGCAG |
| Acsl1 | Forward:GCAACCCCAAAGGAGCAATG |
|  | Reverse:CATCCTCTGGGGAAGCGATG |
| Plin1 | Forward:CCATGTCCCTATCCGATGCC |
|  | Reverse:TCCACCTCTGCTGGAGGATT |
| β-actin | Forward:ATGGATGACGATATCGCTGC |
|  | Reverse:CTTCTGACCCATACCCACCA |
