# Supplementary material for: Molecular mechanism of Cuscutae semen–radix rehmanniae praeparata in relieving reproductive injury of male rats induced with tripterygium wilfordii multiglycosides: A tandem mass tag-based proteomics analysis
Source: Front Pharmacol. 2023 Feb 17;14:1050907. doi: 10.3389/fphar.2023.1050907 (PMC9982038; doi:10.3389/fphar.2023.1050907)
Supplement: Supplementary file 2 [file Table2.docx]

| **Table 2** Effects of Semen Cuscutae–Rehmannia Glutinosa on body weight and testis and epididymal indexes of the experimental rats（$\overline{x}$±s，n = 7） | | | | |
| --- | --- | --- | --- | --- |
| Group | Weight of the 1 week（g） | Weight of the 12 week（g） | Testis index（mg/g） | Epididymal index（mg/g） |
| Control group | 117.57 ±9.78 | 456.86 ±17.70 | 4.02 ±1.76 | 0.88 ±0.13 |
| Model group | 112.57 ±9.34^▲^ | 459.71 ±78.91^▲^ | 3.16 ±0.43^▲^ | 0.77 ±0.11^▲^ |
| TSZSDH group | 109.29 ±3.64^△^ | 491.86 ±24.00^△^ | 3.36 ±0.24^△^ | 0.80 ±0.05^△^ |
