# Supplementary material for: Molecular mechanism of Cuscutae semen–radix rehmanniae praeparata in relieving reproductive injury of male rats induced with tripterygium wilfordii multiglycosides: A tandem mass tag-based proteomics analysis
Source: Front Pharmacol. 2023 Feb 17;14:1050907. doi: 10.3389/fphar.2023.1050907 (PMC9982038; doi:10.3389/fphar.2023.1050907)
Supplement: Supplementary file 3 [file Table5.docx]

| **Table 5** Differential changes in the key proteins of major KEGG pathways | | | | | |
| --- | --- | --- | --- | --- | --- |
| KEGG pathway | Class | Gene name | Fold enrichment | Ratio | -LogP |
| PPAR signaling pathway | Organismal Systems | Acsl1\|Plin1\|Dbil5\|Plin4 | 46.22 | 22.22 | 5.84 |
| Protein digestion and absorption | Organismal Systems | Col12a1\|Col1a1\|Col5a3\|Col1a2 | 38.59 | 22.22 | 5.53 |
| Proteoglycans in cancer | Human Diseases | Dcn\|Col1a1\|Col1a2 | 14.4 | 16.67 | 2.96 |
