# Supplementary material for: Molecular mechanism of Cuscutae semen–radix rehmanniae praeparata in relieving reproductive injury of male rats induced with tripterygium wilfordii multiglycosides: A tandem mass tag-based proteomics analysis
Source: Front Pharmacol. 2023 Feb 17;14:1050907. doi: 10.3389/fphar.2023.1050907 (PMC9982038; doi:10.3389/fphar.2023.1050907)
Supplement: Supplementary file 4 [file Table3.docx]

| **Table 3** Effects of Semen Cuscutae–Rehmannia Glutinosa on the serum sex hormones of the experimental rats | | | | |
| --- | --- | --- | --- | --- |
| Group | LH（mIU/mL） | FSH（ng/mL） | E2（pg/mL） | T（ng/mL） |
| Control group | 148.67 ±13.14 | 58.42 ±21.84 | 320.59 ±58.90 | 0.96 ±0.15 |
| Model group | 139.53 ±22.19^▲^ | 53.85 ±15.14^▲^ | 262.33 ±63.08^▲^ | 0.41 ±0.15^▲^ |
| TSZSDH group | 134.62 ±21.09^△^ | 73.36 ±22.16^△^ | 267.94 ±56.96^△^ | 1.51 ±1.30^☆^ |

Note: ^▲^compared with the control group, model group *P* > 0.05; ^△^compared with model group, TSZSDH group *P* > 0.05; ^☆^compared with model group, TSZSDH group *P* < 0.05.
