# Supplementary material for: Molecular mechanism of Cuscutae semen–radix rehmanniae praeparata in relieving reproductive injury of male rats induced with tripterygium wilfordii multiglycosides: A tandem mass tag-based proteomics analysis
Source: Front Pharmacol. 2023 Feb 17;14:1050907. doi: 10.3389/fphar.2023.1050907 (PMC9982038; doi:10.3389/fphar.2023.1050907)
Supplement: Supplementary file 5 [file Table4.docx]

| **Table 4** Statistics of common differentially expressed proteins between the model and TSZSDH groups | | | | | | |
| --- | --- | --- | --- | --- | --- | --- |
| Number | Protein | Gene | Model group vs.Control group | | TSZSDH group vs.TSZSDH group | |
|  |  |  | P value | Difference fold | P value | Difference fold |
| B2RYW8 | MICOS complex subunit Mic10 | Micos10 | 0.034 | 1.282 | 0.037 | 0.722 |
| P13601 | Aldehyde dehydrogenase, cytosolic 1 | Aldh1a7 | 0.004 | 1.528 | 0.001 | 1.428 |
| A0A0G2JSP8 | Creatine kinase | Ckm | 0.001 | 0.576 | 0.035 | 1.22 |
| A0A0G2K0N6 | Ig-like domain-containing protein | - | 0.016 | 0.627 | 0.007 | 1.793 |
| A0A0G2KAJ7 | Collagen alpha-1(XII) chain | Col12a1 | 0.001 | 0.673 | 0.002 | 1.446 |
| D3ZDF3 | MRV integration site 1 homolog (Mouse) (Predicted), isoform CRA_b | Mrvi1 | 0.028 | 0.394 | 0.049 | 2.221 |
| F1LS40 | Collagen alpha-2(I) chain | Col1a2 | 0.001 | 0.67 | 0.001 | 1.413 |
| F1LSF5 | Perilipin | Plin1 | 0.01 | 0.618 | 0 | 2.983 |
| G3V9M6 | Fibrillin 1 | Fbn1 | 0.017 | 0.788 | 0.01 | 1.338 |
| M0R7S5 | Perilipin 4 | Plin4 | 0.035 | 0.779 | 0.001 | 1.948 |
| P02454 | Collagen alpha-1(I) chain | Col1a1 | 0.001 | 0.602 | 0 | 1.737 |
|  |  |  |  |  |  |  |
| P05545 | Serine protease inhibitor A3K | Serpina3k | 0.005 | 0.427 | 0.001 | 3.395 |
| P06911 | Epididymal-specific lipocalin-5 | Lcn5 | 0.002 | 0.762 | 0 | 8.74 |
| P14141 | Carbonic anhydrase 3 | Ca3 | 0.02 | 0.47 | 0.002 | 4.352 |
| P18163 | Long-chain-fatty-acid--CoA ligase 1 | Acsl1 | 0.004 | 0.743 | 0 | 2.704 |
| P56702 | Diazepam-binding inhibitor-like 5 | Dbil5 | 0.002 | 0.634 | 0.001 | 1.813 |
| Q01129 | Decorin | Dcn | 0.007 | 0.718 | 0.043 | 1.225 |
| Q5BJY9 | Keratin, type I cytoskeletal 18 | Krt18 | 0.017 | 0.699 | 0 | 6.144 |
| Q5GAL8 | Probable inactive ribonuclease-like protein 12 | Rnase12 | 0.048 | 0.757 | 0 | 10.496 |
| Q99ND5 | Sulfotransferase | Sult1e1 | 0.01 | 0.58 | 0.001 | 3.689 |
| Q9JI04 | Alpha 4 type V collagen | Col5a3 | 0.01 | 0.736 | 0 | 1.997 |
| B2RYB1 | Thioredoxin domain-containing 11 | Txndc11 | 0.041 | 0.822 | 0.02 | 1.286 |
